# Advances in Graphene–Polymer Nanocomposite Foams for Electromagnetic Interference Shielding

**DOI:** 10.3390/polym15153235

**Published:** 2023-07-29

**Authors:** Jiaotong Sun, Dan Zhou

**Affiliations:** 1 School of Materials Science and Engineering, Yangtze Normal University, Chongqing 408100, China; zhoudan@yznu.edu.cn; 2 School of Materials Science and Engineering, Chongqing University, Chongqing 400044, China; 3 Chongqing Loncin Industries Co., Ltd., Chongqing 400060, China

**Keywords:** graphene, polymer composites, foam, electromagnetic interference shielding

## Abstract

With the continuous advancement of wireless communication technology, the use of electromagnetic radiation has led to issues such as electromagnetic interference and pollution. To address the problem of electromagnetic radiation, there is a growing need for high-performance electromagnetic shielding materials. Graphene, a unique carbon nanomaterial with a two-dimensional structure and exceptional electrical and mechanical properties, offers advantages such as flexibility, light weight, good chemical stability, and high electromagnetic shielding efficiency. Consequently, it has emerged as an ideal filler in electromagnetic shielding composites, garnering significant attention. In order to meet the requirements of high efficiency and low weight for electromagnetic shielding materials, researchers have explored the use of graphene–polymer nanocomposite foams with a cellular structure. This mini-review provides an overview of the common methods used to prepare graphene–polymer nanocomposite foams and highlights the electromagnetic shielding effectiveness of some representative nanocomposite foams. Additionally, the future prospects for the development of graphene–polymer nanocomposite foams as electromagnetic shielding materials are discussed.

## 1. Introduction

With the increasing convenience and rapid connectivity provided by wireless telecommunication networks, the use of numerous telecommunication devices has resulted in an unprecedented level of electromagnetic interference (EMI). Electromagnetic radiation in the transmission process interferes with other electronic devices via electromagnetic induction, causing the disturbance or malfunction of appliances. Moreover, the growing electromagnetic pollution poses potential risks to human health [1,2]. To tackle this issue, electromagnetic shielding materials are commonly employed to prevent unwanted electromagnetic radiation. These shields usually possess mobile charge carriers to reflect electromagnetic waves, which is the primary route to decrease EMI. Metals with high electrical conductivities are commonly effective EMI shielding materials; however, they are usually dense, prone to corrosion, and difficult to process. Consequently, there is a challenge in developing lightweight, corrosion-resistant, flexible, easy-to-handle, and efficient electromagnetic shielding materials as alternatives to metallic shields.

Polymer nanocomposites attract great attention since they combine the advantages of both polymers and nanoparticles [3,4]. Various nanoparticles with different functions can be dispersed into the polymeric matrix by simple solution/melt blending. Electrically conductive nanofillers such as carbon nanomaterials, nanostructured metals/metal oxides, and 2D transition metal carbides have been used to prepare conductive polymer nanocomposites for effective EMI shielding [5,6,7,8,9,10,11]. In particular, graphene has been widely investigated because of its superior electrical conductivity, extraordinary mechanical properties, and high specific surface area. Its intrinsic conductivity, which is higher than 107 S m^−1^, makes it an ideal nanofiller in polymer nanocomposites for electromagnetic shielding applications [12,13]. Therefore, graphene–polymer nanocomposite films with sufficient electrical conductivity are used for efficient EMI shielding [8,14,15,16,17,18].

When electromagnetic waves contact the surface of the electromagnetic shielding material, electromagnetic radiation will be attenuated in three ways: reflection, the absorption of electromagnetic waves by the shield material, and multiple reflection attenuation inside the shield. Good EMI shielding materials require high electrical conductivity in order to effectively reflect electromagnetic radiation. This is because the shielding material reflects radiation through direct interaction with electromagnetic fields, facilitated by charge carriers. On the other hand, absorption occurs when the radiation interacts with electric or magnetic dipoles in the shielding material. Additionally, scattering at the interface between materials plays a crucial role in the multiple reflection mechanism [1,19]. Therefore, creating heterogeneous structures within the materials can enhance the overall electromagnetic shielding effectiveness of the shield. It is confirmed that the foaming of graphene films improves its EMI shielding efficiency (EMI SE) due to multiple internal reflections by microcellular structures [20,21]. Thus, cellular structures are introduced into graphene–polymer nanocomposites [22]. Foam structures can usually enrich the graphene within or on the cell walls and form electrically conductive networks above the percolation threshold. The resultant graphene–polymer nanocomposite foams have abundant micropores surrounded by a conducting frame, which not only consumes less material but also can achieve a higher EMI shielding performance. Because the porous architectures of the foams can reflect and scatter the incident electromagnetic waves many times between the cell walls, open or closed cells can eventually absorb microwaves after multiple reflections. Meanwhile, since strong EM reflection still causes environmental hazards, it is more environmentally friendly to absorb rather than reflect microwaves.

A range of publications have examined the EMI shielding properties of carbon-based composites [14,15,16,17,18]. These reviews have provided an overview of various composite materials with EMI shielding capabilities when utilizing different carbon nanofillers. This review, however, will specifically concentrate on nanocomposite foams made from graphene and polymers. First, we will summarize the commonly employed techniques used in fabricating graphene–polymer nanocomposite foams. Subsequently, we will detail the preparation methods and EMI shielding performance of several representative graphene–polymer nanocomposite foams. Additionally, we will shed light on the mechanism behind EMI shielding and the factors that enhance the shielding efficiency. Finally, we will offer insights into future advancements in the field of EMI shielding nanocomposite foams.

## 2. Methods for Preparation of Graphene–Polymer Nanocomposite Foams

**Figure 1:**
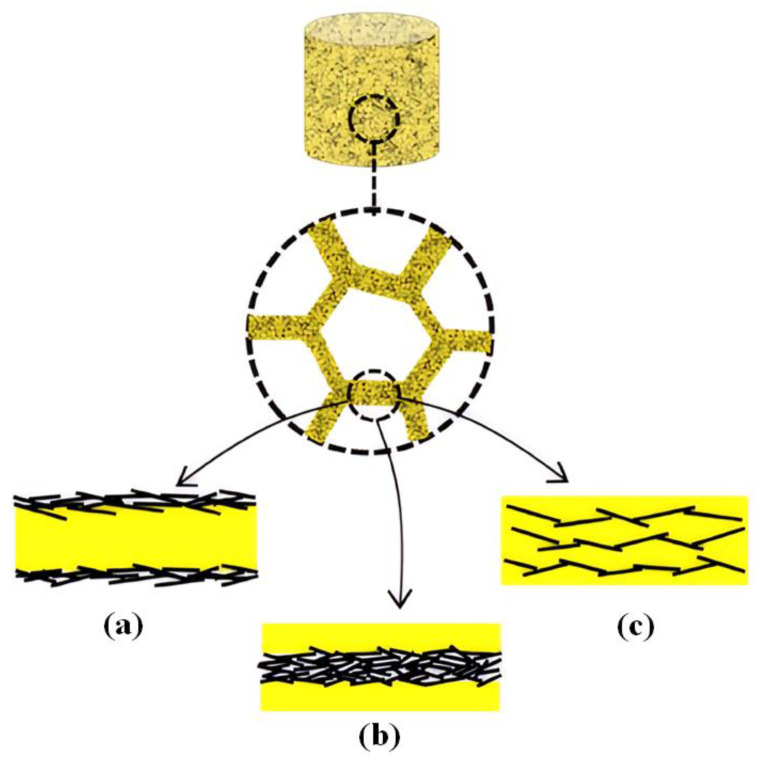
Polymer nanocomposite foams with three different internal structures: (**a**) coating graphene onto polymeric foams; (**b**) graphene foam coated with a polymer layer; (**c**) graphene dispersed in the polymer foam skeleton. Reproduced with permission from [23]. Copyright 2019 Elsevier Ltd.

There are three primary pathways for preparing nanocomposite foams consisting of graphene and a polymer [23]. The internal structures of these foams can be categorized into three types, as shown in Figure 1: (a) polymer foams that are coated with graphene nanosheets; (b) graphene foams that are coated with polymer layers; (c) graphene dispersed within the skeleton of the polymeric foam. The first approach involves using a pre-formed polymeric foam as a template to facilitate the formation of a continuous coating of graphene nanosheets on the foam surface (Figure 1a). The second pathway, on the other hand, entails covering a pre-prepared graphene-based foam with a uniform polymeric coating (Figure 1b). Lastly, an electrically conductive graphene network can be developed within the polymeric skeleton above the percolation threshold, creating a genuine polymer nanocomposite, as depicted in Figure 1c. The detailed procedures available for three different categories are as follows.

### 2.1. Coating Graphene onto Polymeric Foams

**Figure 2:**
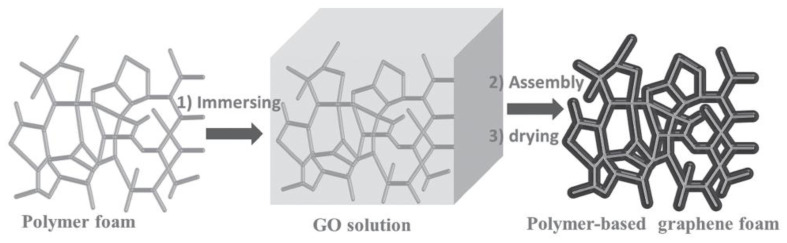
Fabrication process of polymer-based graphene foams. Reproduced with permission from [24]. Copyright 2013 John Wiley & Sons Inc.

**Figure 3:**
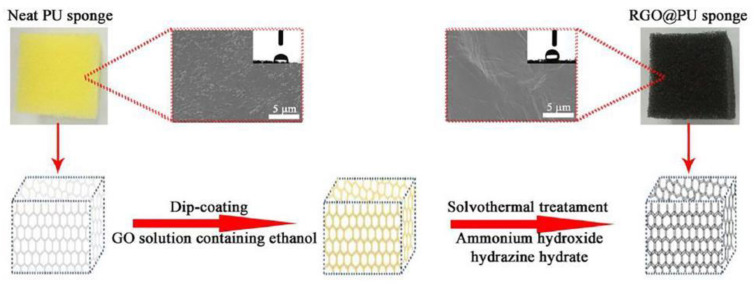
Illustration for preparation of the reduced graphene oxide (RGO)-coated PU sponge. Reproduced with permission from [25]. Copyright 2018 Elsevier Ltd.

The widely used dip-coating method can be facilely implemented to prepare graphene–polymer nanocomposite foams by using polymeric foams as a template [24,25,26,27,28]. The coating of graphene is accomplished by immersing the polymeric foam in an aqueous graphene oxide (GO) nanosheet dispersion and then reducing GO into graphene. Jiang’s group used a commercial polyurethane (PU) foam to fabricate polymer-based graphene foams, as shown in Figure 2 [24]. The process involved immersing the polyurethane (PU) foam into an aqueous solution of graphene oxide (GO) and subsequently chemically reducing it using hydrazine. This resulted in the assembly of hydrophobic graphene nanosheets on the surface of the PU skeleton. The researchers observed a color change in the graphene foams after the pyrolysis of PU, indicating the successful formation of a continuous layer of graphene in a straightforward manner. Xia et al. further modified the process to obtain a PU foam coated with reduced graphene oxide (RGO) [25]. They found that the choice of solvent in the GO dispersion greatly affected the formation of a complete and continuous layer of RGO on the polymer foam surface. To improve the wetting properties of the polymer, ethanol was added to reduce the surface tension of water in the aqueous GO dispersion. This facilitated the assembly of GO on the hydrophobic PU sponge. Finally, the coated GO was reduced through a solvothermal method, as depicted in Figure 3.

### 2.2. Covering Graphene-Based Foams with a Polymer Coating

The second route to graphene–polymer nanocomposite foams is covering the graphene foams with a polymer layer. Graphene foams are typically made by the chemical vapor deposition (CVD) method using a nickel foam (Ni foam) as a template [29]. Since only a few layers of graphene nanosheets are usually produced on the Ni foam surface, the Ni foam should be conserved as a scaffold before further surface modification. The freestanding graphene foams can be obtained by self-assembly and/or the freeze-drying method after the thermal or chemical reduction of GO [21,22,30]. In comparison with a self-assembled graphene foam, the CVD graphene foam has higher electrical conductivity due to its fewer defect sites and the higher quality of its interconnected structure. Meanwhile, the conductivity can be adjusted by changing the layers of grown graphene [29].

**Figure 4:**
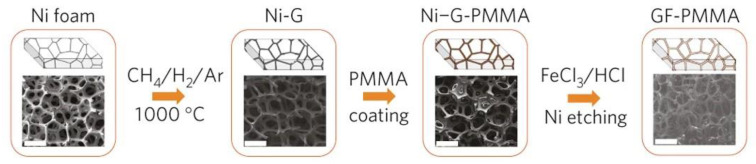
Synthesis of graphene foam (GF) coated with poly(methyl methacrylate) (PMMA). Reproduced with permission from [29]. Copyright 2011 Springer Nature.

**Figure 5:**
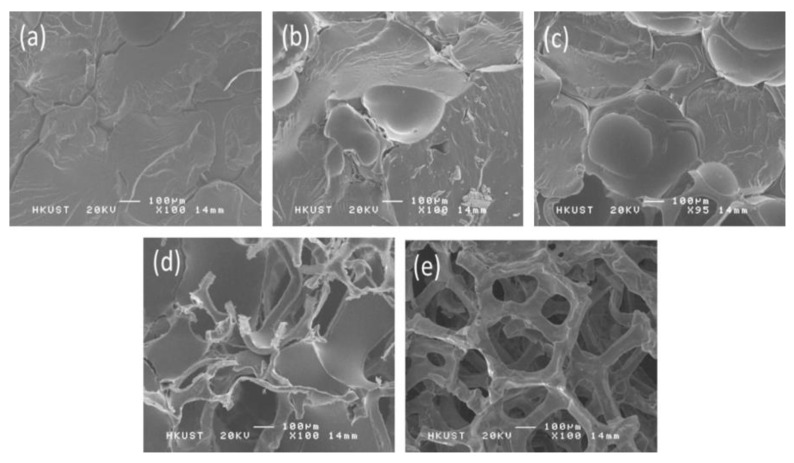
SEM images of graphene foam/PDMS composites with different porosities: (**a**) 9.3%; (**b**) 28.9%; (**c**) 51.5%; (**d**) 73.2%; (**e**) 90.8%. Reproduced with permission from [32]. Copyright 2016 Elsevier Ltd.

For the CVD graphene foam, the Ni foam scaffold is typically treated with a HCl solution after applying a thin polymer layer onto the graphene film (Figure 4). The resulting composite foams made of graphene and polymers exhibit remarkably low densities, below 0.1 g cm^−1^. Coating the graphene foam with a polymeric layer is a straightforward process that involves immersing it in a low-concentration polymer solution [29,31,32,33]. However, in order to maintain the porosity of the graphene foam, it is important not to completely fill the pores with the coating polymer. In one study conducted by Chen et al., the graphene-coated Ni foam was dipped into a dilute solution of Sylgard 184/ethyl acetate for 30 min, followed by curing the silicone layer at a high temperature [31]. Another study by Sun et al. focused on producing graphene foam/poly(dimethyl siloxane) (PDMS) composites with varying densities and porosities by adjusting the weight ratios of PDMS, the curing agent, and the solvent (Figure 5) [32]. It is also possible to apply different polymer layers onto the graphene foam using techniques such as spin coating or in situ polymerization [34,35,36]. By manipulating the duration of the polymerization process, it is simple to control the thickness and morphology of the polymeric layer.

### 2.3. Dispersion of Graphene within Skeleton of Polymer Foams

**Figure 6:**
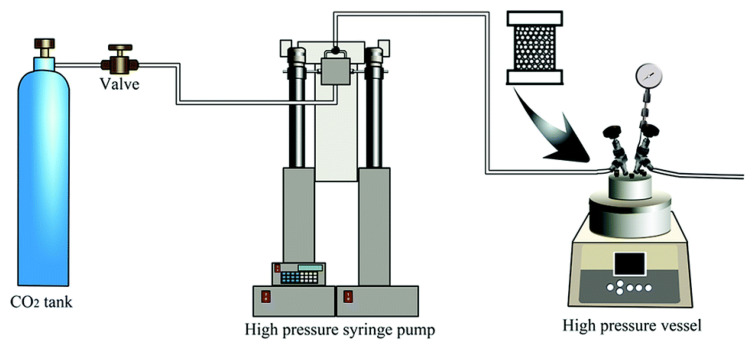
Schematic illustration of the supercritical CO_2_ foaming process [37].

Graphene–polymer nanocomposite foams can also be easily fabricated via a two-step procedure: mixing graphene and polymers to synthesize the nanocomposite and then foaming. The nanocomposites can be obtained by solution mixing or the melt blending of graphene and the polymer, and then the nanocomposite foams are produced by utilizing foaming agents for large-scale production. There are usually two main types of foaming agents: chemical blowing agents and physical blowing agents. Chemical blowing agents include reactive chemicals that produce gases at the decomposition temperature, while physical foaming agents change state from liquid to gas in the foaming process. The most commonly used physical foaming agent is supercritical carbon dioxide (CO_2_) for foam preparation [37]. Briefly, under defined pressure, the polymer nanocomposite sample is first saturated with supercritical CO_2_ at a certain temperature. When the pressure is released, CO_2_ gas bubbles appear and grow, leading to foam formation with the necessary equipment (Figure 6).

Foams with high porosity and a large cell size can be achieved by adjusting certain parameters. These parameters include the dissolution temperature of supercritical CO_2_, the duration of heat treatment, and the rate of pressure release. By manipulating these factors, the size and density of the foam cells can be controlled. More detailed instructions on the foaming process for synthesizing polymer nanocomposite foams can be found in other reviews [38,39,40]. It is important to note that the growth of foam cells affects the spatial arrangement of graphene nanosheets, which in turn influences the interconnection of the nanosheets within the cell walls.

In addition to supercritical CO_2_, chemical foaming agents have also been utilized for the preparation of graphene–polymer nanocomposite foams. For example, in the production of graphene–PU foams, distilled water is used to generate CO_2_ gas through a reaction with isocyanates [41]. Another approach involves the addition of AIBN to pre-prepared nanocomposites, which decomposes and releases nitrogen gas during the compression molding process, resulting in foaming [42]. Similarly, graphene–silicone nanocomposites can be foamed using a reactive process. In this case, the silanes present in polymethylhydrogensilane react with the silanols in hydroxyl-terminated polydimethylsiloxane, generating hydrogen gas as the blowing agent [43].

**Figure 7:**
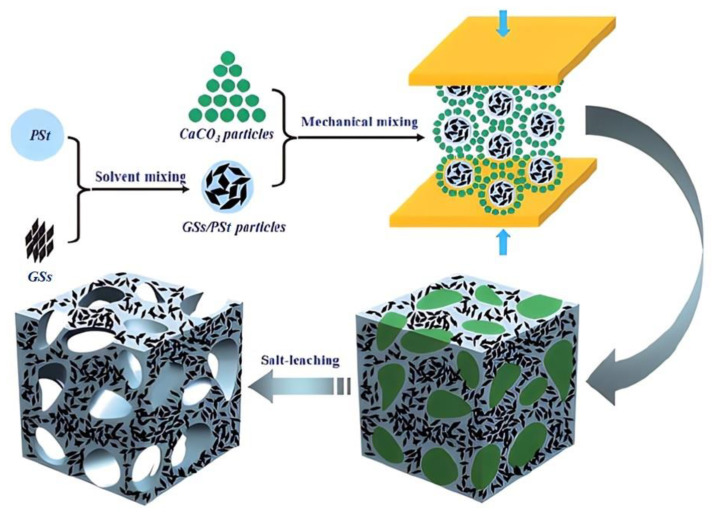
Fabrication of graphene–PSt foam using compression molding and then the salt-leaching method. Reproduced with permission from [47]. Copyright 2012 Royal Society of Chemistry.

In addition to employing blowing agents, phase inversion and leaching are also utilized as methods to fabricate porous polymeric materials. The phase inversion process was first reported to form a polysulfone foam via coagulation of the polymer solution in *N*-methyl-2-pyrrolidone with water vapor [44]. The resultant foam has uniform and closed pores, whose sizes are jointly determined by the solution concentration and the relative humidity. When the latter two parameters both decrease, the pore sizes increase. Zhen et al. employed the method of water-vapor-induced phase separation (WVIPS) to prepare a graphene–polyetherimide (PEI) nanocomposite foam [45,46]. A graphene–PEI dispersion in N, N-dimethylformamide (DMF) was exposed to water vapor with 75% relative humidity at room temperature. The water vapor that diffuses into the DMF dispersion drives phase separation and cell nucleation. Cell growth and hence foam formation cause the preferential accumulation of graphene nanosheets along the cell walls. Yan and coworkers confirmed that the combined methods of compression molding and salt leaching benefit the manufacture of graphene–polystyrene (PSt) nanocomposite foams [47]. Specifically, graphene-sheets (GSs)–PSt microparticles were first prepared and then mechanically mixed with CaCO_3_ microparticles, and they were compression molded together. The porous structure was finally obtained after the dissolution of CaCO_3_ particles in HCl by salt leaching (Figure 7).

## 3. Typical Graphene–Polymer Nanocomposite Foams for EMI Shielding

### 3.1. Graphene–PMMA Nanocomposite Foams

**Figure 8:**
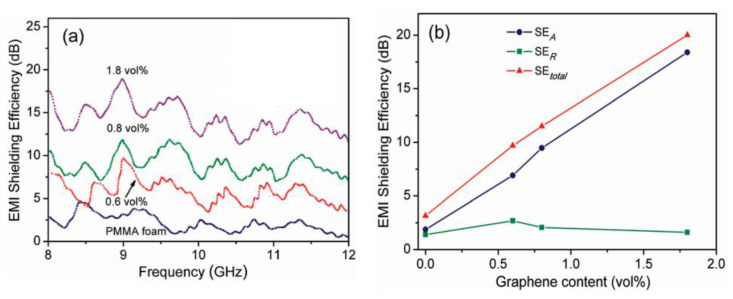
(**a**) EMI SE_total_ of graphene–PMMA nanocomposite foams with different volume contents of graphene from 8 to 12 GHz. (**b**) The values of SE_total_, SE_A_, and SE_R_ at 9 GHz with 0 vol%, 0.6 vol%, 0.8 vol%, and 1.8 vol% graphene. Reproduced with permission from [48]. Copyright 2011 American Chemical Society.

Zhang and coworkers dispersed graphene sheets into a PMMA matrix to fabricate their nanocomposite foams. Microcellular cells with a size dispersity from 1 to 10 μm were made by using subcritical CO_2_ as a foaming agent [48]. The added graphene sheets make the graphene–PMMA foams electrically conductive above the percolation threshold, which is about 0.5 vol% graphene sheets in PMMA foam. Since electrical conductivity greatly affects electromagnetic reflection, their EMI SE is necessarily improved when the content of graphene sheets increases from 0 vol% to 0.6 vol% and further to 1.8 vol%, as shown in Figure 8a. However, as shown in Figure 8b, microwave reflection (SE_R_) contributes less than microwave absorption (SE_A_) to the total shielding efficiency (SE_total_). Specifically, as 1.8 vol% graphene is incorporated, microwave absorption is the dominant mechanism of microwave shielding. This is presumably due to the conductive dissipation of the incident microwave in foam cells with graphene in the foam frame. Multiple reflections can explain the result as a reasonable shielding mechanism. When the incident microwave is frequently reflected and/or scattered by the microcell–frame interfaces, it is difficult for the trapped microwave to escape, and it is eventually absorbed. The specific shielding efficiency (SSE), which is calculated from EMI SE divided by the density, of the composite foam with 1.8 vol% graphene is 17–25 dB cm^3^/g. Meanwhile, a large number of microcells in the foam greatly enhanced the toughness in comparison with its solid counterpart. This work proved that graphene–PMMA microcellular foams would be a light and tough candidate suitable for EMI shielding applications.

### 3.2. Graphene–PSt Nanocomposite Foams

**Figure 9:**
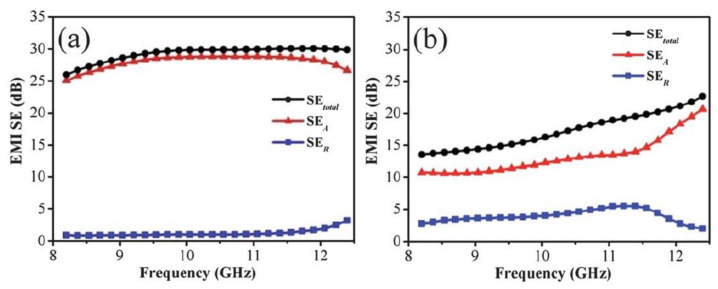
The EMI SE_total_, SE_A_, and SE_R_ of GPS045 (**a**) and GPS027 (**b**) in X-band. Reproduced with permission from [47]. Copyright 2012 Royal Society of Chemistry.

As mentioned before, Yan et al. proposed and implemented a novel method combining compression molding under high pressure and salt leaching to fabricate porous graphene–PSt (GPS) composites [47]. Two products with different porosities and densities were obtained by changing the CaCO_3_ loading. They are labeled GPS045 and GPS027, representing their densities of 0.45 g cm^−3^ and 0.27 g cm^−3^. Correspondingly, their porosities are 60% and 76%. Since the same content (30 wt%) of graphene was loaded, the conductivity of GPS045 (1.25 S m^−1^) was higher than that of GPS027 (0.22 S m^−1^). As shown in Figure 9, the EMI SE_total_ of GPS045 was higher than that of GPS027 in the whole frequency range. The average SE_total_ of GPS045 was approximately 29.3 dB, while that of GPS027 was 17.3 dB for both 2.5 mm thick samples. It indicates that superior conductivity contributes more to the shielding performance than higher porosity. On the other hand, a much greater contribution of SE_A_ to SE_total_ indicates that more microwaves are absorbed through dissipation as heat rather than being reflected. As for this conductive composite foam, electromagnetic absorption is the primary EMI shielding mechanism in the X-band. The SSE of the porous graphene–PSt composite with a thickness of 2.5 mm is surprisingly 64.4 dB cm^3^/g.

### 3.3. Graphene–Polyvinylidene Fluoride (PVDF) Nanocomposite Foams

**Figure 10:**
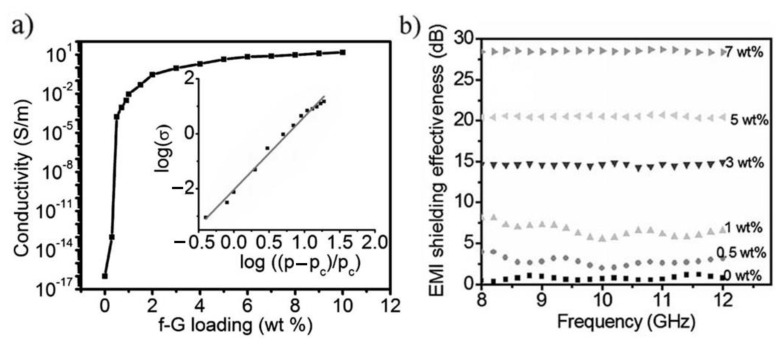
(**a**) The conductivity of composite foams versus weight percentage of loaded f-G. Inset: plot of log conductivity versus log (p − p_c_)/p_c_ for the composite foams. (**b**) EMI SE for f-G/PVDF foam composites in X-band. Reproduced with permission from [42]. Copyright 2011 John Wiley & Sons Inc.

Eswaraiah et al. first functionalized graphene with a H_2_SO_4_ and HNO_3_ mixture and then mixed functionalized graphene (f-G) with PVDF and the foaming agent (2, 2′-azobisisobutyronitrile) in dimethyl formamide (DMF). The composite film was casted in a Petri dish and dried in the oven. The f-G/PVDF foam with cell sizes of 0.5–2 μm was prepared by hot pressing due to the decomposition of the foaming agent [42]. The effect of f-G content on the conductivity and EMI shielding effectiveness (EMI SE) of the composite foam was investigated (Figure 10). With the increasing mass fraction of f-G, the conductivity increases sharply from insulating 10^−16^ S m^−1^ for neat PVDF to conducting 10.16 S m^−1^ for the PVDF composite foam with 2 wt% f-G. The f-G fillers form conductive paths throughout the PVDF matrix above the percolation threshold (p_c_ = 0.5 wt%) (Figure 10a). The highest EMI SE was 28 dB in the X-band region for the foam composite with 7 wt% f-G (Figure 10b). In contrast to the former graphene–PMMA and graphene–PSt composite foams, the f-G/PVDF foam showed a reflection-dominant shielding mechanism. Its reflectivity and absorptivity were 78% and 21%, respectively, and this result is consistent with that of the pure graphene film. Therefore, it is possible that the smaller and fewer pores in these composite foams could not play the same role as those in the former graphene–PMMA and graphene–PSt composite foams.

Recently, a binary polymer matrix of PVDF and epoxy resin (EP) was used to disperse and fix graphene nanosheets (GNS) functionalized with nickel–cobalt (Ni-Co) alloy particles to form high-performance EMI shielding composites [49]. The graphene nanosheet with Ni-Co particles (GNS-Ni-Co) was first made by an in situ growth method on acidulated GNS surfaces. The salt-leaching method was employed to prepare 3D porous GNS-Ni-Co/PVDF composites with NaCl as a salt template. The resultant composites were dipped in epoxy resin and cured to obtain GNS-Ni-Co/PVDF/EP composites. When GNS-Ni-Co content increased, SE_T_ and SE_A_ both increased, but SE_R_ changed little. The incident electromagnetic waves were attenuated by multiple internal reflections, absorption, and scattering due to the heterogeneous structure of hybrid fillers and the porous structure of the composites.

### 3.4. Graphene–PEI Nanocomposite Foams

**Figure 11:**
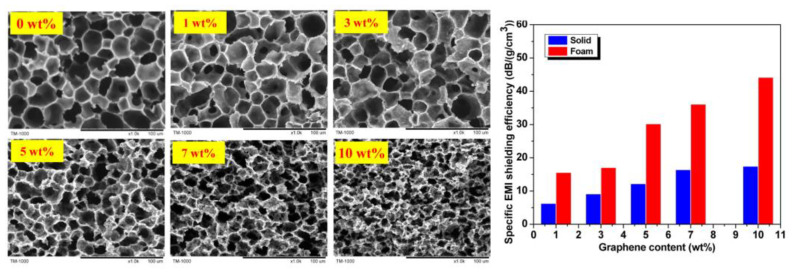
SEM images of graphene–PEI nanocomposite foams at different graphene loadings (**left**); The EMI SSE of graphene–PEI nanocomposite solids and foams at 9.6 GHz (**right**). Reproduced with permission from [45]. Copyright 2013 American Chemical Society.

Zhai et al. employed the WVIPS method to facilely prepare graphene–PEI nanocomposite foams [45]. The homogenized graphene–PEI nanocomposite in DMF was exposed to preset humidity and temperature, and then a 2.3 mm thick foam sheet with uniform cells was obtained. When the graphene content was below 3 wt%, the foam cells exhibited a diameter of around 16 μm (Figure 11). With an increase in graphene loading, the cell size gradually decreased due to higher viscosity in the graphene–PEI dispersion and the increased hindrance of cell coalescence caused by the presence of more graphene. Even at 10% graphene loading, the cell size was reduced to 9.0 μm, while all foam samples maintained a consistent density of 0.3 g/cm^3^. Transitioning from the solid nanocomposite structure to the foam structure led to a decrease in the percolation threshold from 0.21 vol% to 0.18 vol%. This could be attributed to the growth of cells that facilitated the flow of graphene sheets, resulting in the orientation and accumulation of graphene along the cell walls. At the same loading of graphene (10 wt%), the electrical conductivity was 4.8 × 10^−6^ S/cm for graphene–PEI nanocomposite solids, while for the foam counterpart, it was 2.2 × 10^−5^ S/cm. Additionally, foaming significantly increased the EMI SSE from 17 to 44 dB/(g/cm^3^). These graphene–PEI nanocomposite foams also demonstrated a high Young’s modulus and extremely low thermal conductivity.

Zhai and coworkers further developed a high-performance graphene@Fe_3_O_4_ (G@Fe_3_O_4_)/PEI composite foam by introducing Fe_3_O_4_ nanoparticles into the graphene–PEI system [46]. The flexible composite foam was prepared facilely by using the same WVIPS method, and it also had the advantage of low density (0.28–0.4 g/cm^3^). The incorporated Fe_3_O_4_ nanoparticles improved impedance matching and attenuated the incident electromagnetic wave. The microwave was thus eventually absorbed via the combination of multiple reflections. The as-prepared G@Fe_3_O_4_/PEI foam with 10 wt% content of G@Fe_3_O_4_ had an EMI SSE as high as ∼41.5 dB/(g/cm^3^) in the X-band region. Meanwhile, the introduction of Fe_3_O_4_ nanoparticles endowed the resulting foams with superparamagnetic behavior, which facilitated its use in EMI shielding and magnetic actuation applications.

### 3.5. Graphene–Polyimide (PI) Nanocomposite Foams

**Figure 12:**
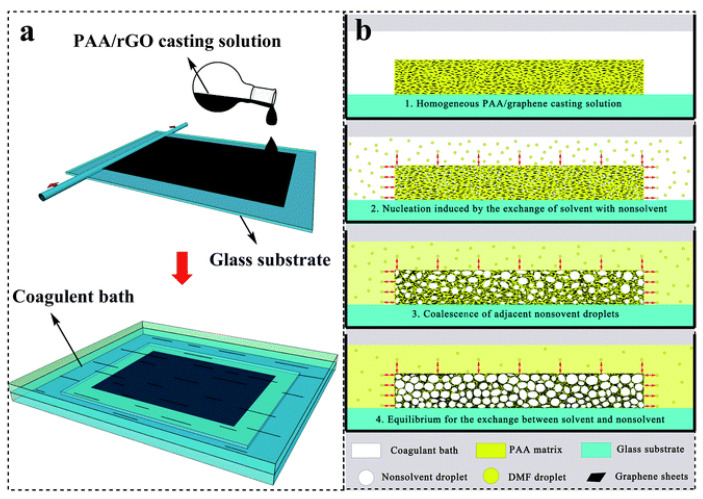
Schematic operation for nonsolvent-induced phase separation (NIPS) by soaking the casting film of PAA/rGO in a coagulant bath (**a**) and the formation process of PAA/rGO composite foams (**b**). Reproduced with permission from [50]. Copyright 2015 Royal Society of Chemistry.

The heatproof aromatic polyimide (PI) was also used to prepare a graphene composite foam in three steps [50]. The polycondensation of 4,4′-diaminopheyl ether and pyromellitic dianhydride in the presence of reduced GO (rGO) gave birth to poly(amic acid) (PAA)-modified rGO. Then, the rGO/PAA composite foams were prepared by casting the rGO/PAA solution and soaking it in a nonsolvent (alcohol/water mixture) bath (Figure 12). The rGO/PI composite foams were obtained after thermal imidization and tested for EMI shielding. The lightweight rGO/PI composite foam with 16 wt% rGO showed an EMI SE of 17–21 dB at 8–12 GHz with a thickness of 0.8 mm. The thermostability and mechanical properties of the foam remained good in comparison with those of the PI solid, which helped pave the way for its practical application in the electronics industry.

In contrast to the previous approach of directly using reduced graphene oxide (rGO), Yang et al. implemented a different method to create lightweight rGO/PI composite foams [51]. They initiated the process by preparing a poly(amic acid) (PAA) solution using N,N-dimethylacetamide (DMAc) with dispersed graphene oxide (GO). They then induced phase separation by utilizing dibutyl phthalate as a nonsolvent to produce porous composite films. Subsequently, the porous films were subjected to heat treatment to simultaneously convert PAA and GO into polyimide (PI) and rGO through thermal imidization and thermal reduction. Various measurements were conducted to validate the conversions. The resulting porous PI/rGO film, with a thickness of 500 μm and 8 wt% rGO, exhibited an electrical conductivity of 0.015 Sm^−1^ and an impressive electromagnetic interference specific shielding effectiveness (EMI SSE) of 693 dB cm^−2^ g^−1^. Similar to the previous study, the incorporation of PI into the composite film provided exceptional thermal stability and mechanical properties.

Li and coworkers prepared a PI open-cell foam with pyromellitic diester and catalysts as component A and polyphenylene isocyanate as component B by a free-foaming method [52]. Apart from the different foaming means, the graphene nanoplatelets and Fe_3_O_4_ nanoparticles were both adsorbed onto the PI foam by dip coating. The impregnated PI foam with graphene–Fe_3_O_4_ fillers exhibited an EMI SE of 60.6 dB at a thickness of 6.0 mm with low reflection. When the loading of Fe_3_O_4_ nanoparticles increased, the conductivity and EMI SE of composites rose as well due to the synergistic effect of graphene and magnetic Fe_3_O_4_ nanoparticles. Additionally, dilute graphene could reduce reflections on the external surface, make more microwaves enter the interior, and realize the absorption domination of microwaves.

### 3.6. Graphene–PU Nanocomposite Foams

**Figure 13:**
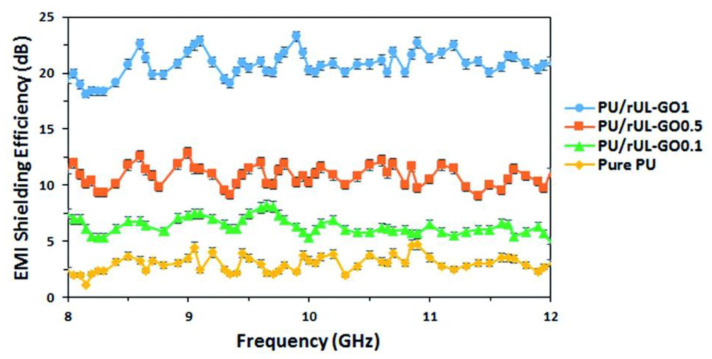
EMI shielding efficiency of different PU/rUL-GO composite foams at different frequencies. Reproduced with permission from [41]. Copyright 2016 Royal Society of Chemistry.

Graphene–PU foams have been made by different groups using different methods due to the unique properties of the PU matrix. Gudarzi and coworkers fabricated multifunctional graphene–PU composite foams via in situ polymerization with reduced ultralarge graphene oxide (rUL-GO) [41]. The addition of 1 wt% rUL-GO turned the PU insulator into a composite conductor with an electrical conductivity of 4.04 S m^−1^. The relationship curves of the EMI shielding efficiency versus frequency for composite foams with the incorporation of different rUL-GO are shown in Figure 13. A low percolation threshold and an EMI SSE of 253 dB (g^−1^ cm^−3^) at 8–12 GHz were achieved due to the method of foam preparation and a uniform dispersion, together with a high aspect ratio for rUL-GO. The introduced rUL-GO improved the mechanical properties of the PU matrix without a decline in flexibility.

**Figure 14:**
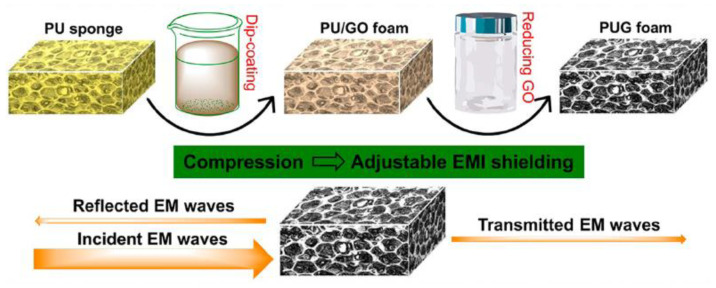
Overall fabrication process of PUG foams, including dip coating GO sheets onto the PU framework and then hydrothermally reducing them with hydrazine vapor. Reproduced with permission from [53]. Copyright 2016 American Chemical Society.

Zheng et al. facilely fabricated compressible and lightweight graphene–PU foams by dip coating commercial PU sponges in a graphene oxide aqueous suspension, followed by the hydrothermal reduction of coated GO in the presence of hydrazine monohydrate [53]. The resultant graphene–PU foams possessed a low density of 0.027–0.030 g/cm^3^. More importantly, they showed high-performance EMI shielding efficiency, which was possibly contributed to by an absorption-dominant mechanism, conductive dissipation, multiple reflections, and the scattering of EM waves by the conductive graphene network (Figure 14). Benefiting from great compressibility, their EMI SE is adjustable and can be easily adjusted by compression.

Lin and colleagues conducted a study in which they utilized a dip-coating technique to prepare PU foams coated with Ni-rGO. They used nickel sulfate in a GO aqueous suspension during the process [54]. Excess sodium borohydride was employed to reduce GO and nickel sulfate, resulting in the formation of rGO and Ni nanoparticles on the surface of the PU sponge. The resulting Ni-rGO-coated PU foams, with a thickness of 10 mm, exhibited an impressive electromagnetic interference shielding effectiveness (EMI SE) of 24.03–27.71 dB within the frequency range of 30–1500 MHz. These foams successfully met the practical requirements while maintaining the original compressibility of the PU foams.

### 3.7. Graphene–PDMS Nanocomposite Foam

**Figure 15:**
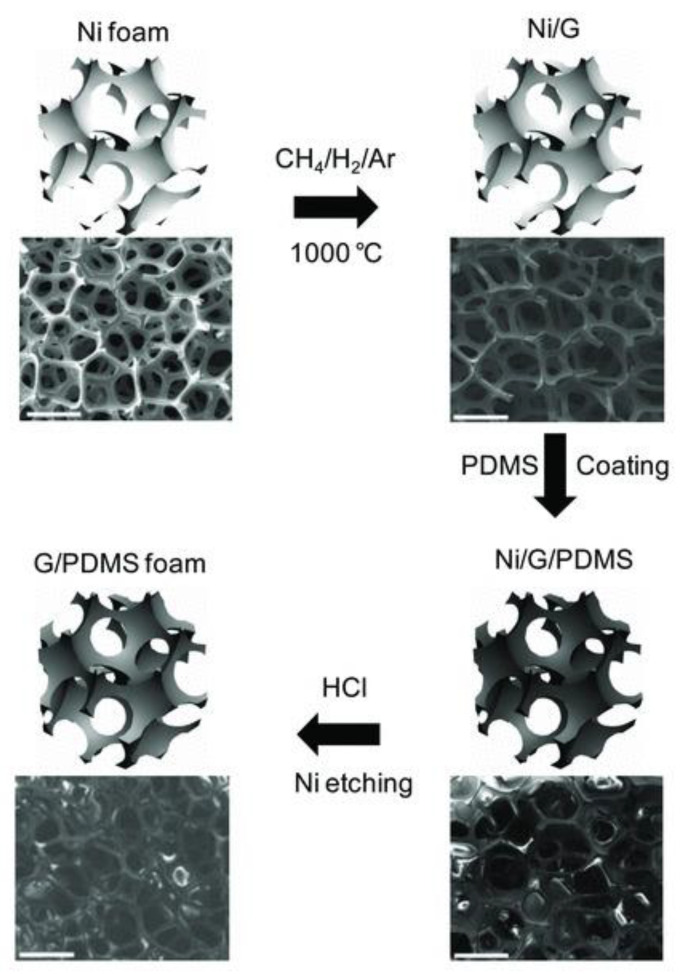
Schematic of the procedure for fabricating graphene–PDMS foam composites. The scale bars are 500 μm. Reproduced with permission from [31]. Copyright 2013 John Wiley & Sons Inc.

Chen et al. introduced a new method to overcome the limitations of chemical-derived graphene, which often exhibits poor electrical conductivity and high inter-sheet contact resistance, in 2013. They successfully grew graphene on a nickel foam by using the chemical vapor deposition (CVD) of methane at 1000 °C [31]. To enhance the quality and electrical properties of graphene, a thin layer of PDMS (polydimethylsiloxane) was applied to its surface through a dip-coating process. Subsequently, HCl was used to etch away the nickel substrate, resulting in the fabrication of a graphene–PDMS nanocomposite foam. The simplified fabrication steps are depicted in Figure 15.

**Figure 16:**
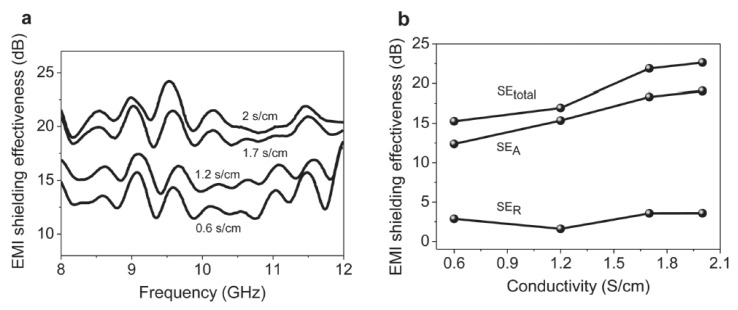
(**a**) EMI shielding effectiveness of graphene–PDMS foam composites with different electrical conductivities measured in the frequency range of 8–12 GHz (X-band). (**b**) Comparison of SEtotal, SEA, and SER of graphene–PDMS foam composites with different electrical conductivities at a frequency of 9 GHz. Reproduced with permission from [31]. Copyright 2013 John Wiley & Sons Inc.

The composites were fabricated without the use of foaming agents, and the prepared graphene sheets in the composite foams were seamlessly interconnected. The graphene content and the electrical conductivity of the composite could be tuned by changing the conditions in the CVD process. The graphene–PDMS foam formed had a low density of 0.06 g/cm^3^ at a low graphene content of <0.8 wt%, and its EMI SE reached 30 dB with a specific EMI SE of up to 500 dB · cm^3^/g (Figure 16). It also could be seen that both the SE_total_ and SE_A_ increased, while SE_R_ remained almost unchanged when the electrical conductivity increased. It is worth noting that absorption contributed more to the EMI shielding effectiveness than reflection. Moreover, the graphene–PDMS foam composites showed excellent flexibility and stable shielding effectiveness after bending 10,000 times. This lightweight and highly conductive graphene–PDMS nanocomposite foam would have high performance in EMI shielding applications.

**Figure 17:**
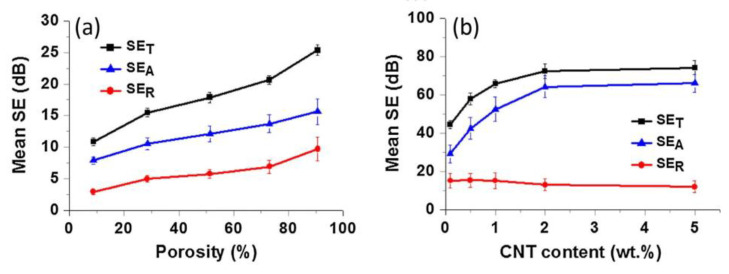
Comparison of mean values of SE_T_, SE_A_, and SE_R_ over the whole frequency range of (**a**) GF/PDMS composites with different porosities and (**b**) GF/CNT/PDMS composites with different CNT contents. Reproduced with permission from [32]. Copyright 2016 Elsevier Ltd.

Sun et al. created cellular-structured composites made of PDMS and porous graphene foams (GFs) and conductive nanoscale carbon nanotubes (CNTs) [32]. These multi-scale hybrid composites possess inherent percolation and a high porosity of 90.8%, which results in a remarkable EMI SE of 75 dB. This is a 200% enhancement compared to composites made solely from GFs with the same graphene content and porosity (Figure 17). The EMI SSE is 833 dB cm^3^/g, which is one of the highest values reported for all carbon filler–polymer composites. The hybrid reinforcement structure of the composites creates significant synergy between the GFs and CNTs. The GFs drive microwaves to be attenuated by the dissipation of the currents induced by electromagnetic fields, while the CNTs expand the conductive networks and introduce numerous interfaces with the matrix, thus enhancing the dissipation of surface currents.

Wang et al. designed and prepared porous composites with polyaniline-modified melamine foam as a framework, Fe_3_O_4_/rGO as fillers, and PDMS as a binder to anchor the fillers onto the framework by dip coating [55]. With Ag-plated aramid paper as the bottom, the asymmetric conductive structure was formed. The conductive polyaniline and Fe_3_O_4_/rGO on the skeleton synergistically gained excellent impedance matching and electromagnetic absorption performance, while the conductive Ag formed on the bottom helped the asymmetric composite achieve a superior EMI SE of 70 dB and a high absorption coefficient of 0.86 in the X-band.

### 3.8. Graphene–Poly(3,4-ethylenedioxythiophene):Poly(Styrene Sulfonate) (PEDOT:PSS) Nanocomposite Foams

**Figure 18:**
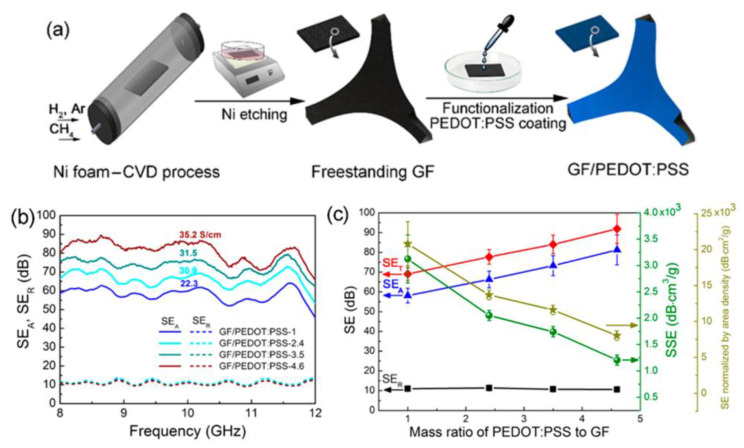
(**a**) Schematic procedure for the preparation of GF/PEDOT:PSS composites; EMI SEs of composites with different compositions: (**b**) SE_A_ and SE_R_ and (**c**) summary of SEs, SSEs, and SSEs normalized by area density as a function of mass ratio of PEDOT:PSS to GF. Reproduced with permission from [56]. Copyright 2017 American Chemical Society.

Graphene–poly(3,4ethylenedioxythiophene):poly(styrenesulfonate) (PEDOT:PSS) foam composites were prepared by drop coating PEDOT:PSS on freestanding graphene foams (GFs) [56]. GFs were also fabricated by the CVD process on a Ni foam and then Ni etching. To improve their wettability and enhance the interfacial bonds with PEDOT:PSS, the freestanding GFs were first functionalized with 4-dodecylbenzenesulfonic acid. The graphene–PEDOT:PSS composite foams showed a low density of 18.2 × 10^−3^ g/cm^3^ and a high porosity of 98.8%. The electrical conductivity was enhanced from 11.8 to 43.2 S/cm after the incorporation of the conductive PEDOT:PSS. The composites had an incredible EMI SE of 91.9 dB and a high SSE of 3124 dB·cm^3^/g because of their high electrical conductivity, porous structure, and effective charge delocalization (Figure 18). The excellent electrical conductivities and left-handed composites with absolute permittivity and/or permeability larger than one probably gave rise to significant microwave attenuation by absorption.

### 3.9. Graphene–Poly(arylene ether nitrile) (PEN) Nanocomposite Foams

Zhang and coworkers recently reported a porous absorption-dominated EMI shielding material composed of poly(arylene ether nitrile) (PEN), graphene–carbon nanotubes, and Fe_3_O_4_ particles [57]. The composite foam was obtained by soaking the casting film of PEN/iron ions/graphene/carbon nanotubes in DMAc/NH_4_ · H_2_O via nonsolvent-induced phase separation (NIPS). The Fe_3_O_4_ particles were grown in situ in NH_4_ · H_2_O by the co-precipitation method. The incorporated graphene–carbon nanotubes enhanced the conductive loss of incident microwaves, while the magnetic particles contributed to the dielectric loss and magnetic loss. When the content of Fe_3_O_4_ was 3.55 wt%, the composite foam had the highest EMI SE of 38 dB and the highest absorption ratio of 94%. The PEN matrix also rendered good thermostability for the composite foam, which paved the way for its practical application in EMI shielding.

## 4. Conclusions and Outlook

This mini-review does not aim to provide a comprehensive summary of all the recent progress made in this field, as there has been an increasing amount of research and continuous presentation of smarter designs with better results. Several good reviews have already provided different perspectives on related topics, such as the foaming of polymeric composites and the structural properties of effective EMI shielding materials [58,59,60,61,62,63,64,65]. In this review, we focus on frequently used preparation methods and highlight several typical graphene–polymer nanocomposite foams that have been developed in recent years. The field of graphene-based nanocomposite foams as electromagnetic shielding materials has seen great progress, both in the exploration of new preparation methods and in the improvement of electromagnetic shielding effectiveness. The current challenge in the EMI field is to develop high-efficiency, light, thin, strong, and broadband electromagnetic shielding materials. To achieve this goal, different approaches can be attempted, including the following:

(1) Enhancing the EMI shielding effectiveness of polymers: Although polymers have advantages such as light weight, corrosion resistance, and easy processing, most polymers have weak intrinsic shielding properties against electromagnetic waves. To enhance their EMI shielding effectiveness, conductive polymers such as polyaniline and polypyrrole can be selected as EMI shielding scaffolds.
(2) Incorporating other functional materials: Adding metallic powders, semiconductors, inorganic/organic compounds, and magnetic nanoparticles can improve impedance matching and increase the dissipation capacity of electromagnetic waves. Additionally, new two-dimensional materials such as oxides, nitrides, and black phosphorus can be added as impedance amplitude modulators and media to improve the impedance matching of materials and enhance polarization and multiple reflections at the interface.
(3) Investigating the role of porosity and cell size: Although some studies have mentioned the influence of porosity and cell size on the EMI shielding effectiveness of composite foams, a systematic investigation is still needed to understand the general rules governing how and why the cell density and size affect the reflection or absorption of incident electromagnetic waves.
(4) Considering thickness-specific efficiency: Currently, the weight-specific shielding efficiency of foams is often emphasized, while the thickness-specific efficiency is usually ignored. It would be more reasonable to evaluate the shielding efficiency by dividing it by the thickness dimension of samples.

With rational design and effective implementation, graphene-based foam materials are expected to play a significant role in electromagnetic shielding due to their ultralight weight, high strength, and exceptional efficiency.

## Data Availability

Not applicable.
